# Performance and Durability of Thin Film Thermocouple Array on a Porous Electrode

**DOI:** 10.3390/s16091329

**Published:** 2016-08-23

**Authors:** Erdogan Guk, Manoj Ranaweera, Vijay Venkatesan, Jung-Sik Kim

**Affiliations:** Aeronautical & Automotive Engineering Department, Loughborough University, Loughborough LE11 3TU, UK; E.Guk@lboro.ac.uk (E.G.); R.A.M.P.Ranaweera@lboro.ac.uk (M.R.); V.Venkatesan@lboro.ac.uk (V.V.)

**Keywords:** solid oxide fuel cell, thin-film thermocouples, temperature measurement, film characterisation

## Abstract

Management of solid oxide fuel cell (SOFC) thermal gradients is vital to limit thermal expansion mismatch and thermal stress. However, owing to harsh operation conditions of SOFCs and limited available space in stack configuration, the number of techniques available to obtain temperature distribution from the cell surface is limited. The authors previously developed and studied a thermocouple array pattern to detect surface temperature distribution on an SOFC in open circuit conditions. In this study, the performance in terms of mechanical durability and oxidation state of the thin film thermoelements of the thermocouple array on the porous SOFC cathode is investigated. A thin-film multi-junction thermocouple array was sputter deposited using a magnetron sputter coater. Scanning electron microscopy (SEM) and X-ray photoelectron spectroscopy (XPS) characterisation techniques were carried out to understand characteristics of the thin film before and after temperature (20 °C–800 °C) measurement. Temperature readings from the sensor agreed well with the closely placed commercial thermocouple during heating segments. However, a sensor failure occurred at around 350 °C during the cooling segment. The SEM and XPS tests revealed cracks on the thin film thermoelements and oxidation to the film thickness direction.

## 1. Introduction

Solid oxide fuel cell (SOFC) technologies have received significant attention as an attractive potential candidate to overcome future energy problems due to the high conversion efficiency achieved, typically above 50% [1]. High operating temperatures allow SOFCs to use hydrocarbons as fuel without requiring noble catalysts such as platinum or palladium [2]. However, mechanical durability and degradation caused by high operating temperatures stand as fundamental barriers to SOFC performance and prevent the complete commercialization of SOFC at industrial scales [3]. Owing to limited available techniques, temperature distribution of SOFC cell electrodes has not been understood fully.

Therefore, the measurement of surface temperatures in SOFCs is a necessary requirement in order to better understand SOFC temperature gradients along the cell surface, in turn aiding in overcoming degradation issues. Understanding thermal gradients on the cell electrode offers opportunity to able to limit differential thermal expansion and induced thermal stress [4]. Even though a considerable amount of research on understanding the temperature distribution of SOFC is available in the literature, a large proportion of the research is focused on computer simulation or modelling to estimate the temperature profile of a cells’ surface [5,6,7,8]. The lack of experimental studies is mainly owing to limitations in available techniques, harsh operating environment and limited space for implanting additional devices within SOFC systems.

The authors’ previous study [9] has addressed the available techniques for experimental measurement of SOFC cell surface temperature during operation, along with their limitations. Thermocouples are generally preferred as a convenient and implementable technique for harsh SOFC operating conditions [7,10,11,12]. However, thermocouples are widely used to measure the inlet and outlet gas temperature [13,14]. A recently developed thin film thermocouple array was used for SOFC temperature measurement directly from the cell surface [9,15]. A thin film thermocouple array provides two significant advantages over the traditionally available thermocouple. Firstly, it decreases the number of thermoelements used to create the sensing points due to its architecture. Secondly, it increases the spatial and temporal resolution due to being in thin film form and less distortion to environment [10,16,17]. However, during the cooling segment, at a temperature around 600 °C, sensor failure had occurred [15]. It was reasoned that this was due to connection disruption between external wires and thin film thermoelements pads.

In this study, the performance of the thin film thermocouple array fabricated directly on a porous substrate was investigated at a temperature range from 20 °C to 800 °C. The sensors then are examined by SEM and XPS to obtain information about the physical durability and oxidation of the thin film thermoelements themselves. XPS is a widely used technique to analyse surface chemistry and to measure elemental composition, chemical and electronic state within a material.

## 2. Experimental Details

### 2.1. Material Selection and Sensor Fabrication

A thin film thermocouple array sensor was fabricated on the cathode surface of a Next-Cell electrolyte-supported cell (Fuel Cell Materials) cathode surface. Standard K-type thermocouple materials alumel (500 nm) (Ni:Mn:Al:Si/95:2:2:1 by wt.) and chromel (500 nm) (Ni:Cr/90:10 by wt.) were the thermoelement materials employed. K-type thermocouples offer sufficient temperature range to cover the operating temperature of a typical SOFC (from 600 °C to 900 °C). The cathode, used as the substrate to fabricate sensors, has a porous structure with a thickness of 50 µm while the total cell thickness of the cell is approximately 0.17–0.2 mm (0.17 mm electrolyte and 50 µm anode electrodes), which offers enough strength to handle the substrate during both the sensor deposition and the annealing process.

Sputtering was selected as a deposition technique to fabricate the thin film thermoelements on the cell cathode surface. Sputtering for thin film deposition is widely used due to low deposition temperature, uniform film deposition and easy process controllability [18]. A Q150T S sputter coater was used for sputtering with the parameters shown in Table 1. Alumel (Ni:Mn:Al:Si/95:2:2:1 by wt.) and chromel (Ni:Cr/90:10 by wt.) were used as target materials.

**Figure 1:**
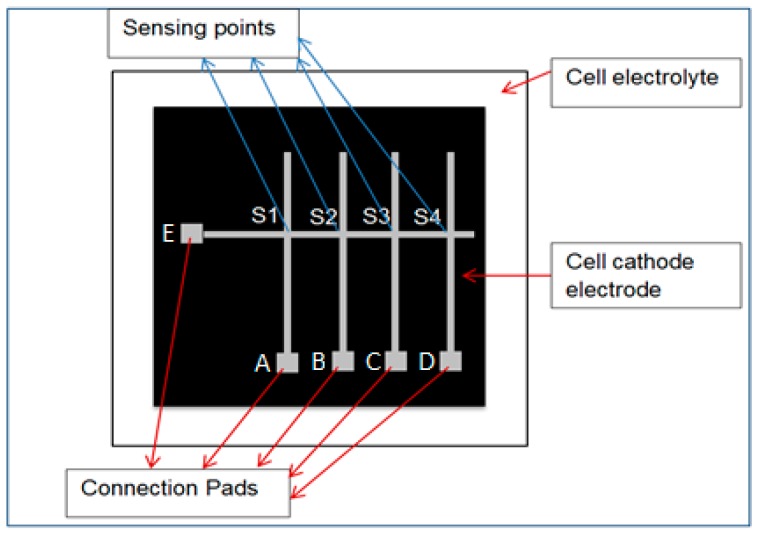
Schematic view of sputter-deposited 500 nm thick sensor thin film thermocouple array architecture.

Four alumel and one chromel thermoelements were deposited separately by using a patterned metal mask. The mask is made of metal (302-stainless steel) and was designed for alumel thermoelements with four slits and chromel thermoelements with one slit. A single horizontal line of chromel thermoelement (E) was fabricated, along with four corresponding vertical alumel thermoelements (A, B, C, D), yielding four sensing points (S1, S2, S3, S4), as depicted in Figure 1. The size (area) of thin film thermoelements were enlarged at the points A, B, C, D and E, designated as thin film pads in order to have enough surface area for external wire attachments. The resulting array provided four sensing points using five thermoelements only; for comparison, commercial thermocouples would have required eight thermoelements for the same number of sensing points [15].

### 2.2. External Wires Connection

**Figure 2:**
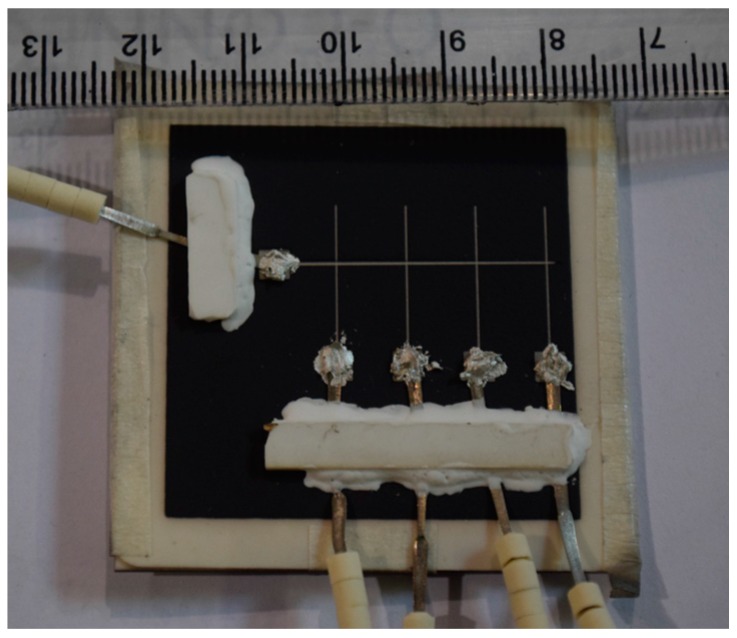
External wires attached to the thin film thermoelement pads.

Four Alumel external wires (Φ 0.5 mm) were connected to thin film pads A, B, C and D, and a chromel external wire (Φ 0.5 mm) was connected to thin film pad E to form K-type thermocouple junctions (see Figure 1 for pad positions and Figure 2 for connections). Silver paste (Aremco-bond 597) was applied to provide electrical connections between the pads and external wires, and additionally possesses excellent electrical conductivity up to 900 °C. However, silver paste is not capable of carrying the mechanical load induced by the wires attached to it. Therefore, alumina strips, connected with alumina paste, were used to increase the mechanical strength of the connection and minimize unwanted external loads from wires and beads depicted in the Figure 2. The alumina was cured at 250 °C for 2 h, and the silver paste was cured at 90 °C for 1 h.

Two commercial thermocouples (K-type) were also placed very close to the substrate surface. Temperatures from the thin-film multi-junction thermocouple, as well as those from the commercial thermocouples were recorded at 1 Hz using an NI 9213 data logger and a LabVIEW based data logging system. 

## 3. Results and Discussion

### 3.1. Temperature Reading

**Figure 3:**
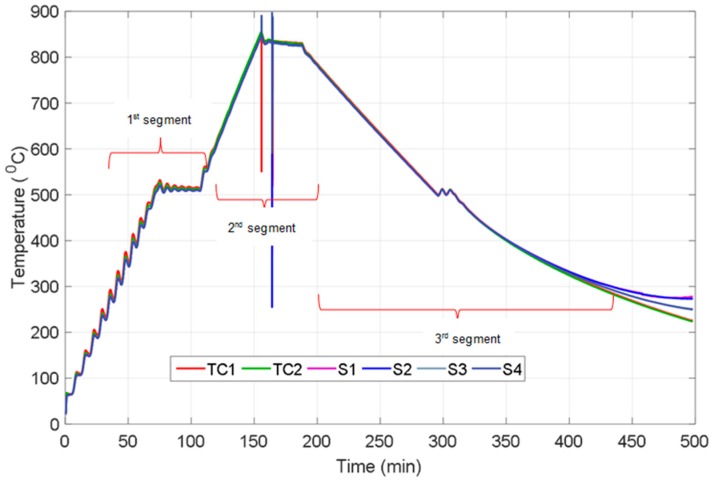
Temperature profile of two thermocouples and four sensing point of the sensor.

The temperature reading from thermocouple array and commercial thermocouples were obtained and plotted as seen in the Figure 3 for a given heating segments. From Figure 3, it can be found that the temperature reading from the thermocouple array has good agreement with the commercial thermocouples during the 1st segments from room temperature to 500 °C. The minor temperature fluctuations from room temperature to 500 °C were produced by controlling the heating pattern of the furnace to monitor sensor performance during sudden and repeating variations in temperature. As depicted in Figure 3, measurements from the sensors exhibit same behaviour with the two K-type thermocouples and reflect even small variations in temperature. In the second segment, in which a continuous heating was introduced, the furnace temperature was increased from 500 °C to around 800 °C. The measurements from the two thermocouples and the sensor readings from four sensing points produced excellent agreement in this step.

During the 3rd segment from 800 °C to 500 °C, all the sensing points and thermocouples follow exactly the same trend, which coincide with/overlap over each other and almost appear to be a single line; this was followed by another dwelling phase at 500 °C for 30 min. The thermocouples and sensor points continued to follow the same trend down to 350 °C during cooling; however, there is a diversion that starts after this temperature as shown in Figure 3. The two thermocouples kept slightly decreasing whilst the temperature readings from the sensing points S1, S2 and S3 stopped their decrease and levelled out. Despite measurements from the sensing point S4 continuing to decrease, it was not similar to the decrease observed in the thermocouples. After 500 min the temperature reading from the all sensing points diverged from each other (see Figure 3) and there was no reading obtained from the sensor while the thermocouples continued to provide correct readings down to room temperature. It is proposed that the reason leading to sensor failure is a high degree of resistance in the thermoelements or at the connection point between external wires and thin film thermoelements pads, which is connected by silver paste.

Table 2 shows difference between the electrical resistances of each of the sensing points before and after the heating cycle. As is clearly seen from Table 2, there is a drastic increase from 20 Ω to 6 MΩ in the resistance of the sensing points. This significant rise in resistance leads variations in temperature readings resulting in drift due to the metallurgical changes of the thermoelements [17]. Oxidation of the thin film or external wire, thin film cracking, poor electrical contact or delamination and cracks in the silver paste during cooling were considered as the reasons for the sharp increase in resistance, which resulted in the failure of temperature measurement [17]. Thermal expansion mismatch (between silver paste, external wires and substrate material at the connection point) causes cracking in, and removal of, silver paste during cooling due to the tensile stress. SEM and XPS were carried out to examine the failed thin film sensor, and the results are presented and discussed in the following section.

### 3.2. Film Characterisation Tests

#### 3.2.1. SEM Analysis

**Figure 4:**
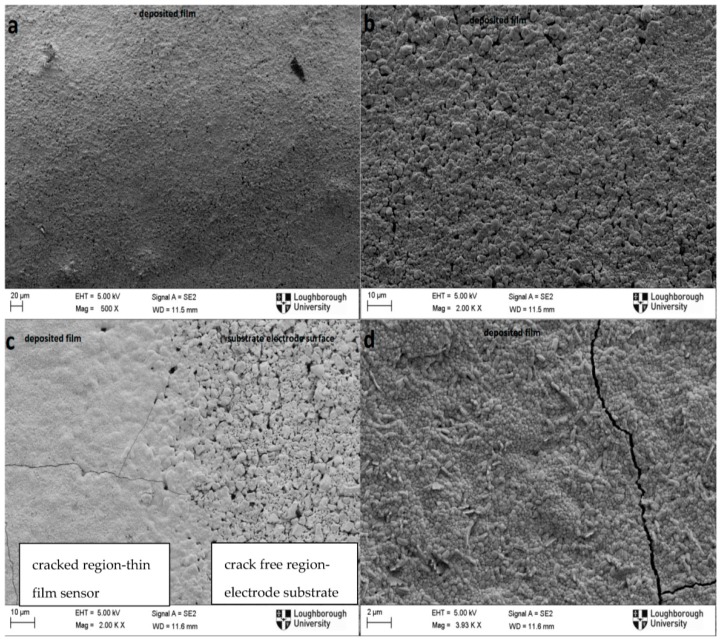
SEM images of 500 nm thick thin film thermoelements before (**a**,**b**) and after (**c**,**d**) thermal cycling at 800 °C.

Figure 4 shows the SEM images of the longitudinal view of thin film thermoelements under different magnification before (Figure 4a,b), and after (Figure 4c,d) the experiment. Figure 4a,b indicate that there were no microcracks generated on the thin film sensor during sputtering, and hence no cracking was present before the heating cycles. Transverse micro-scale cracks were observed after thermal cycling as seen from Figure 4c,d). It is worth noting that cracks only occurred on the deposited sensor film while there was no cracking observed on the substrate (the cathode) nearby the deposited film (Figure 4c). Thermal expansion mismatches [17,19] and different friction rate [20,21] between the deposited thin film material and the substrate material are the main reasons proposed for the crack formed on the thin film. Due to the higher thermal expansion coefficient of the fabricated thin film thermoelements (metal), the thin film thermoelements (metal), experience more shrinkage than the support materials (ceramic) due to the higher thermal expansion coefficient of the fabricated thin film thermoelements (metal). This in turn creates tensile stress, resulting in transverse and longitudinal cracks on the thin film thermoelement as shown in Figure 4c,d.

Cracks due to the tensile stress occurring on the surface of the ceramic during cooling cycles are more prone to propagate compared to the cracks created due to compression loading during heating cycle [19,22]. It is highly possible that the formed cracks will negatively contribute to the electrical potential drop between the two thermoelements, leading to significant increase in resistance; even so, it is not considered as a primary reason for signal losses due to the available path for electrical conduction throughout the thermoelements.

#### 3.2.2. XPS Analysis

XPS depth profiling analysis from surface to bulk was carried out to investigate the oxidation state of one of the thin film thermoelements of the sensor before and after annealing in air at 800 °C. The etching rate was 0.15 nm/s for 50 min (450 nm thickness). Nickel as dominant material (95%) and Oxygen in the thermoelements are destructively analysed before and after the heating cycle.

**Figure 5:**
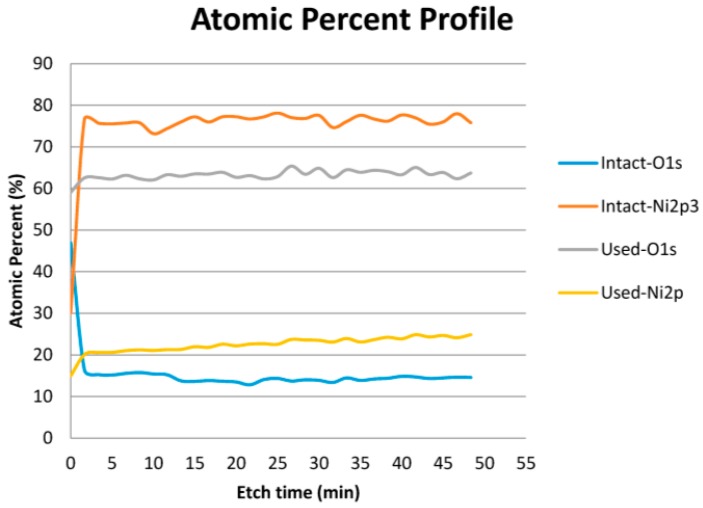
Atomic percentage profile of nickel and oxygen before and after the experiment.

As seen from Figure 5, XPS analysis of the top surface (~1 nm depth) of the thin film layer revealed considerable changes in the percentage of Ni and O_2_ before and after the heating cycle. As expected, the atomic percentage of nickel increased drastically from 30% to around 78%, while oxygen atomic percentage decreases from 48% to about 15% after 2 min etching and both stay at same level during the etching process for the intact sample. This is due to surface (1–5 nm) oxidation of nickel based thermoelements at room temperature [23]. The possible reason for the oxygen (15%), seen through film thickness, is the reasonably porous structure of the film (see Figure 4b) allowing oxygen penetration. On the other hand, there is no significant change revealed in the oxygen and nickel percentage between the surface and the thin film layer during etching for the used thermoelements. Atomic percentage of both nickel and oxygen increase by roughly 5% after 2 min of the etching process and remain stable throughout the etching process for the heat treated samples. A slight increase in nickel with increase in etching depth is observed due to decrease in oxygen penetration with etching depth. Eventually, the thin film is oxidised significantly during heating cycle, leading to an increase in resistance (see Table 2) [17]. Oxidation is one of the most important reasons for chemical degradation even for the ceramic materials in thin film thermoelements [24]. As is shown from the XPS result, the thin film thermoelements materials (Ni) oxidised significantly.

There are two important disruptions that oxidation can cause, especially in metal thin film thermocouples. Firstly, it can lead to drift [10,25] due to chemical changes in the material composition of the thermoelements, which has small effect (0.2–0.5 °C/h) on the temperature reading under 1000 °C [24]. Drift, due to the metallurgical time-dependant changes in the thermocouple array thermoelements, leads to incorrect readings being obtained over time. Secondly, rapid increase in the oxidation rate at high temperature cause morphology changes and leads to bubbling, resulting in delamination [10]. In this study, it is proven from the results obtained from the characterisation techniques (SEM and XPS) that insulation, or more stable thermoelements such as ceramic based materials [17,26], is a crucial requirement and is vital to protect thin films from not only oxidation but also chemical interaction with the substrate material [27]. However, oxidation and micro-scale cracks are not evaluated as the real reason for sensor failure due to their relatively light impact on the temperature reading compared to connection-related issues. Thus, it is reasoned that high resistance between the external wires, silver paste and thin film pad at the connection point is the primary mechanism leading to sensor failure [25].

## 4. Conclusions

In this study, a recently developed thin film (500 nm) thermocouple sensor architecture, which has potential applications for SOFC temperature sensing, was fabricated on an SOFC cell cathode electrode and tested in air at 800 °C. Temperature readings from the four sensing points were obtained. Temperature readings from the two commercially available thermocouples (placed nearby the sensing points) and those from the sensor were generally in agreement throughout heating segments down to 350 °C during the cooling segment. Sensor failure occurred at around 350 °C resulting in divergent readings henceforth.

Significant difference was noticed between the resistances measured before and after the experiment for each sensing point (S1–S4). High resistance is proposed as the main reason for sensor failure. SEM and XPS characterisation techniques were performed to understand main source for high resistance and the failure mechanism of the sensor. As a result of characterisation tests, significant transverse cracking and high oxidation throughout thin film layer were obtained. Even though micro-scale cracks and oxidation state contribute negatively and lead to drift in temperature readings, these are not considered as main sources for sensor failure. The resistance at the connection pads between external wires, thin film pad and silver paste is proposed as a more convincing and impactful reason for sensor failure. In the next study, an insulation layer will be applied to protect thermoelements from the harsh operating environment, and will be investigated under SOFC test conditions.

## Figures and Tables

**Table 1:** Sputtering parameters.

Temperature	<50 °C
Deposition rate	~14 nm/min
Current	140 mA

**Table 2:** Measured resistance of four sensing points before and after annealing process.

Electrical Resistance	S1	S2	S3	S4
**Before (Ω)**	20	25	30	37
**After (MΩ)**	6	8	1	2

## Supplementary Materials

The supplementary materials are available online at http://www.mdpi.com/1424-8220/16/9/1329/s1.
