# Supplementary material for: Performance and Durability of Thin Film Thermocouple Array on a Porous Electrode
Source: Sensors (Basel). 2016 Aug 23;16(9):1329. doi: 10.3390/s16091329 (PMC5038619; doi:10.3390/s16091329)
Supplement: Supplementary file 1 [file sensors-16-01329-s001.pdf]

Supplementary Materials: Performance and Durability of Thin Film Thermocouple Array on a Porous Electrode

Erdogan Guk, Manoj Ranaweera, Vijay Venkatesan and Jung-Sik Kim *

Figure S1 shows OCV values under varying temperature. A thin film thermocouple array sensor was fabricated on the cathode surface of a Next-Cell electrolyte-supported cell (Fuel Cell Materials) cathode surface. Standard K-type thermocouple materials alumel (500 nm) (Ni:Mn:Al:Si/95:2:2:1 by wt.) and chromel (500 nm) (Ni:Cr/90:10 by wt.) were the thermoelement materials employed. The pattern layout is described in Figure 1 of the manuscript. In addition, two commercial K type thermocouples were placed in close proximity (~2 mm from cathode surface). Hydrogen was used as fuel at the anode side while the cathode side was under open atmosphere. The temperature of the furnace is increased manually to observe the relationship between OCV and temperature readings. It was shown that the OCV decreases with the increase in temperature, and it is in agreement with the Nernst equation. The results show that both the commercial thermocouples and the thin film sensor array followed the same trends, but displayed differential temperature readings. S4 and the thermocouples were in close agreement (~5 °C variance) whilst S1 and S2 showed the greatest difference between the thermocouples and the sensor temperature readings. This acknowledges the presence of significant temperature gradients within a small geometric distance, and it is appropriate that S1 and S2 show similar readings as they are adjacent to each other. Likewise, S4 shows close correlation with the thermocouples. The causes attributable to temperature gradients on the cell surface are reduced to (i) proximity to the heating elements within the furnace; and (ii) localised chemical exothermic/endothermic reactions.

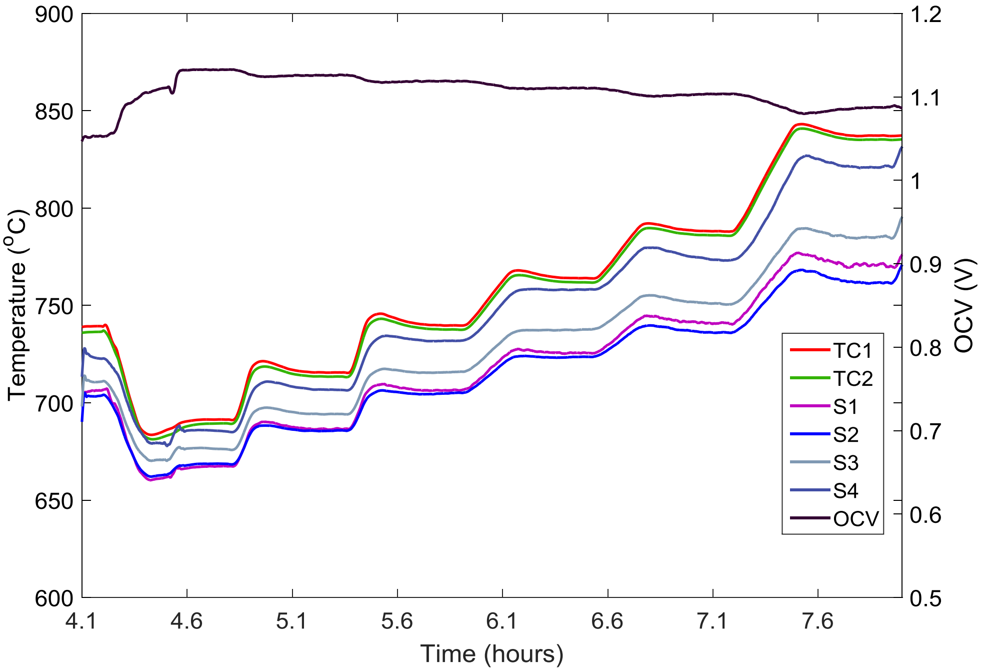

Figure S1. OCV variation with temperature from two commercial thermocouples and four sensing points of thin film array.

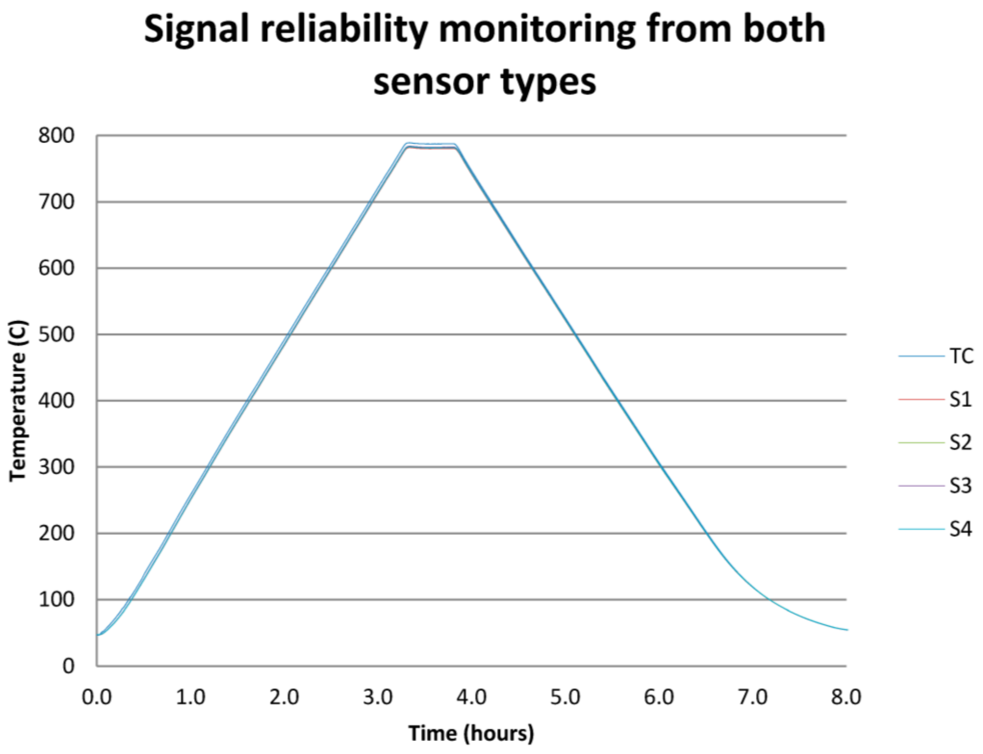

Figure S2. A sensor signal comparison between an encased thin film array and a commercial K type thermocouple over a single heating cycle.

An alumina-encased thin film array was put through a signal reliability test on the same SOFC cell with an improved connection technique. The connection issues were dealt with by utilising a spring based system in conjunction with gold plating to ensure constant mechanical contact between connection elements (wires, pad, and silver paste). The connecting interfaces, namely the end of the connecting wire and the pads on the sensor array were sputter-coated with gold to protect against oxidation. Conductive silver paste is applied between these two elements.

The alumina encasing is achieved by sputtering the cell cathode surface with 100 nm of aluminium, and then subjected to an oxidising step at 900 °C. This is followed by sputtering the sensor array pattern replicated as per Figure 1 in the manuscript, employing alumel (500 nm) (Ni:Mn:Al:Si/95:2:2:1 by wt.) and chromel (500 nm) (Ni:Cr/90:10 by wt. Finally, a 20 nm layer of aluminium is sputter deposited and subjected to the oxidation regime as per the previous layer.

The test consisted of subjecting the cell to a heating cycle, without fuel supply. The result as shown in the Figure S2 demonstrates the improved resilience of the sensor array as well as the connection mechanisms, as there is no loss of signal transmission throughout both the heating and cooling segments. Furthermore, there is much closer agreement between sensor array readings and the commercial thermocouples, potentially yielding more accurate results during practical operation temperature monitoring.
